# Prognostic Value of PD-L1, BAP-1 and ILK in Pleural Mesothelioma

**DOI:** 10.3390/jcm13237322

**Published:** 2024-12-02

**Authors:** Oliver Illini, Michal Benej, Anna Sophie Lang-Stöberl, Hannah Fabikan, Luka Brcic, Florian Sucher, Dagmar Krenbek, Tibor Krajc, Christoph Weinlinger, Maximilian J. Hochmair, Arschang Valipour, Thomas Klikovits, Stefan Watzka

**Affiliations:** 1 Department of Respiratory and Critical Care Medicine, Clinic Floridsdorf, Vienna Healthcare Group, 1130 Vienna, Austria; 2 Karl Landsteiner Institute of Lung Research and Pulmonary Oncology, Clinic Floridsdorf, 1210 Vienna, Austria; 3 Department of Thoracic Surgery, Clinic Floridsdorf, Vienna Healthcare Group, 1210 Vienna, Austria; 4 Karl Landsteiner Institute for Clinical and Translational Thoracic Surgery Research, Clinic Floridsdorf, 1210 Vienna, Austria; 5 Diagnostic and Research Institute of Pathology, Medical University of Graz, 8010 Graz, Austria; 6 Department of Pathology, Clinic Floridsdorf, Vienna Healthcare Group, 1210 Vienna, Austria; 7 Paracelsus Medical University, 5020 Salzburg, Austria

**Keywords:** pleural mesothelioma, prognostic biomarkers, PD-L1, BAP-1, ILK, multimodality treatment

## Abstract

**Background**: Pleural mesothelioma (PM) is a rare type of cancer with poor prognosis. Prognostic and predictive biomarkers could improve treatment strategies in these patients. Programmed death ligand 1 (PD-L1), integrin-linked kinase (ILK) and breast cancer gene 1-associated protein (BAP-1) have been proposed to predict outcomes in PM, but existing data are limited and controversial. **Design and Methods:** This single-center, retrospective study analyzed data on expression patterns and the prognostic role of PD-L1, ILK and BAP-1 in consecutive patients diagnosed with PM. **Results:** Of all patients (n = 52) included, more than half showed a positive PD-L1 expression (52% TPS ≥ 1%, 65% CPS ≥ 1), 69% showed a BAP-1 loss and 80% an ILK ≥ 50%. Positive PD-L1 expression was more frequent in the non-epithelioid subtype (*p* = 0.045). ILK intensity (*p* = 0.032) and positive PD-L1 (*p* = 0.034) were associated with more advanced tumor stages. The median overall survival (OS) was 16.9 (95% CI 13.1–25.2) months. Multimodality therapy (MMT) including surgery and early stage were independent prognostic factors for longer OS (MMT: HR 0.347, 95% CI 0.13–0.90, *p* = 0.029; advanced stage: HR 4.989; 95% CI 1.64–15.13, *p* = 0.005). Patients with an expression of PD-L1 TPS ≥ 1% or BAP-1 positivity showed numerically worse survival with a median OS of 15.3 (11.5; 24.4) vs. 20.0 (11.2; 34.9) and 11.3 (5.6; 31.0) vs. 20.0 (15.2; 28.1) months, respectively. Furthermore, PD-L1 was associated with worse survival in patients receiving MMT (PD-L1 TPS ≥ 1%: 15.8 (12.1–25.4) vs. 31.3 (17.4–95.4) *p* = 0.053). ILK expression ≥50% did not influence survival. The combinations of CPS ≥ 1% with BAP-1 positivity or ILK expression ≥50% were associated with worse survival (*p* = 0.045, *p* = 0.019). **Conclusions:** In this real-world analysis, expressions of PD-L1 and BAP-1 were associated with worse survival in patients with PM. ILK showed no prognostic value. Further studies with larger cohorts are needed to identify prognostic and predictive biomarkers facilitating optimized individual treatment decision in this rare type of cancer.

## 1. Introduction

Pleural mesothelioma (PM) is a rare but aggressive type of cancer that affects the mesothelial cells of the pleural lining and is associated with asbestos exposure in over 80% of cases [1,2]. Although most types of asbestos were banned in the European Union in 1991, the decreasing use of asbestos has only resulted in a stabilization of the age-standardized incidence rates, which range between 0.7 to 1.4 per 100,000 persons in Europe due to the fact that PM has a latency period of 30 to 56 years between exposure and onset [1,3,4,5,6]. Furthermore, there are still numerous countries in Asia that continue to mine, import, and use asbestos despite its hazardous impact [7]. Before onset, patients suffer from pleural inflammation for several years, until a transformation into PM occurs [8]. Upon presentation, PM has a very poor prognosis with median overall survival (OS) ranging from 12 to 30 months for localized disease and from 8 to 14 months in an advanced stage [9]. A high-risk factor for PM development is a pathogenic germline mutation in the tumor suppressor gene Breast Cancer gene 1-associated protein (BAP-1), which can cause a predisposition for mesothelioma and an increased sensitivity to asbestos exposure [10]. Prognostic indicators can help predict likely survival outcomes in patients. While the European Organization for Research and Treatment of Cancer (EORTC) proposed that poor performance status, leukocytosis, sarcomatoid histologic type and male gender are predictive of a poor prognosis, more recently, several biomarkers have been discussed to be associated with PM prognosis [11,12,13]. A potential biomarker is integrin-linked kinase (ILK), which has been linked to chronic inflammation leading to remodeling of the extracellular matrix and has been detected in human PM samples, while not appearing in normal mesothelial cells or normal lung parenchyma [14]. Another promising biomarker is the programmed death ligand 1 (PD-L1), which leads to the inhibition of apoptosis of tumor cells and promotes T-cell exhaustion [15]. While PD-L1 expression has been found to correlate with a favorable prognosis in some cancers, it was shown to be a negative prognostic factor in PM as well as renal and gastric cancers [16,17,18]. However, most studies assessing the prognostic value of PD-L1 did not include patients treated with immunotherapy (IO) [15,16,19,20]. IO, on the other hand, combining PD-1 and cytotoxic T-lymphocyte-associated protein 4 (CTLA-4) antibodies, has shown promising survival benefits for patients with PM in recent studies [21,22]. Nevertheless, limited and controversial data exists on the prognostic role of BAP-1, ILK and PD-L1 expression in predicting outcomes in PM [8,15,16,19,23,24,25,26,27]. Therefore, this study aims to investigate the expression patterns of BAP-1, ILK and PD-L1 and their correlation with overall survival time. 

## 2. Materials and Methods

### 2.1. Study Design

This was a retrospective single-center analysis of patients with a histologically confirmed PM treated at the Department of Thoracic Surgery and the Department of Respiratory and Critical Care Medicine at the Clinic Floridsdorf in Vienna, Austria. The aim of this study was to analyze clinical data of patients with PM and to investigate the expression patterns of BAP-1, ILK and PD-L1 and their correlation with OS. 

This study was reviewed and approved by the ethics committee of the city of Vienna (EK 14-030-VK), Austria, and conducted in accordance with the Declaration of Helsinki of the World Medical Association [28].

### 2.2. Patients

Medical records of patients with PM diagnosed between January 2010 and December 2021 were reviewed. Patients were included if they were over the age of 18 and had a histologically confirmed diagnosis of PM, adequate clinical data and a sufficient amount of formalin-fixed paraffin-embedded (FFPE) tumor tissue from diagnostic procedures or surgery. Histological verification was based on the 2021 WHO classification of tumors of the pleura [29]. Tumor staging was performed according to the 7th TNM classification [30]. After inclusion, predefined clinical data on patients’ age, gender, clinical stage, histologic subtype, treatment and survival for each patient was collected in an anonymized form in a dataset for statistical analysis. FFPE samples of each patient were sent for immunohistochemical staining and analysis to the Diagnostic and Research Institute of Pathology, Medical University of Graz, Austria. 

### 2.3. Pathological Studies

**Figure 1:**
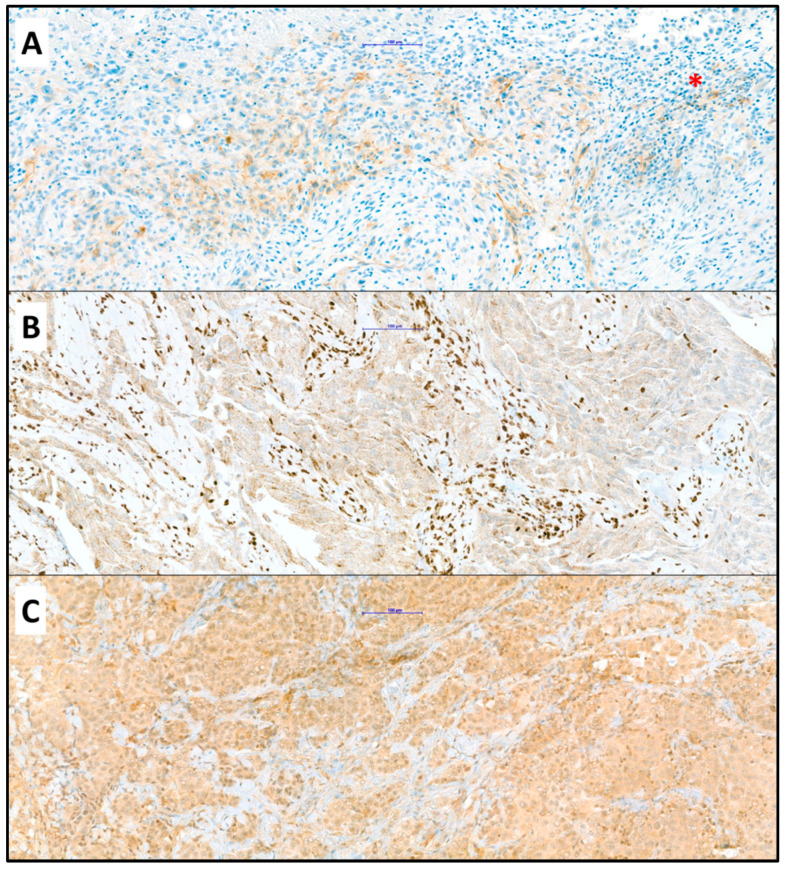
(**A**) Positive reaction of mesothelioma cells to antibody against PD-L1 with few positive lymphocytes (asterisk); (**B**) Loss of nuclear staining in mesothelioma cells with unspecific granular cytoplasmic reaction and clearly positive nuclear staining of lymphocytes (positive internal control); (**C**) Positive reaction of mesothelioma cells to antibody against ILK; (Objective ×20, immunohistochemistry). The blue bar indicates 100 µm.

Histopathological specimens were acquired either during diagnostic procedures or surgery. Tissue samples were routinely processed and diagnostically examined according to standard of care. PM was confirmed by histopathological analysis of hematoxylin and eosin-stained 4 µm slides and categorized as PM of epithelioid, sarcomatoid or biphasic histologic subtype [29]. Further immunohistochemical analyses of BAP-1, ILK and PD-L1 expression were conducted at the Diagnostic and Research Institute of Pathology, Medical University of Graz (Figure 1). We used BAP1 Clone C-4 (Santa Cruz, Dallas, Texas, US, sc-28383, dilution 1:100), PD-L1 Clone SP263 (Ventana, Roche, Rotkreuz, Switzerland, ready to use) and ILK phospho S246 (abcam, Cambridge, UK, ab111435, dilution 1:100). PD-L1 expression was determined by both the tumor proportion score (TPS) and the combined positive score (CPS). The TPS assesses the percentage of ≥100 viable tumor cells that show complete or partial PD-L1 staining at any intensity, while the CPS considers the expression of PD-L1 in both tumor and inflammatory cells, expressed in relation to all available tumor cells [31]. Histological specimens were considered to have PD-L1 expression if TPS ≥ 1% or CPS ≥ 1. ILK expression was considered positive if cells with ILK expression were detected in ≥50% of tumor cells. BAP-1 was evaluated as positive or negative (loss) nuclear reaction. 

### 2.4. Treatment

Due to the long duration of this study, diagnosis and treatment were conducted in each patient according to the respective current ESMO Clinical Practice Guidelines for PM [32]. After diagnosis and tissue sampling, patients were treated with either multimodality treatment including surgery (MMT), active treatment but not MMT or best supportive care (BSC). MMT was defined as the combination of surgery by either pleurectomy/decortication (P/D), extended pleurectomy/decortication (EPD) or extrapleural pneumonectomy (EPP) with either chemo-/immune-/and or radiotherapy. The type of surgery depends on each patient’s clinical presentation and disease characteristics. To preserve the lung, preferably EPD or a P/D was performed. EPP was performed when both pleura and lung parenchyma were affected. Patients received adjuvant radiotherapy if the spatial distribution of the disease allowed exclusive pleural and mediastinal radiotherapy without excessive damage to the preserved lung parenchyma. Patients not responding to neoadjuvant chemotherapy or unsuitable for surgery underwent subsequent radiotherapy if feasible, plus follow-up or the best supportive care only. Instead of neoadjuvant chemotherapy, adjuvant chemotherapy following pathological diagnosis was administered if PM was not suspected at the time of surgery [33]. Active oncologic treatment but no MMT consisted of either platinum-based chemotherapy (CHT), immunotherapy (IO), palliative radiotherapy (RT), combined chemoradiotherapy (RT-CHT) or only surgery. 

### 2.5. Statistical Analysis

All data were analyzed with Stata version 14 (StataCorp LP, College Station, TX, USA) and figures were created using Stata version 14 (StataCorp LP, College Station, TX, USA) and SigmaPlot version 11.0 (Systat Software Inc., Franktfurt am Main, Germany). Data distribution was tested for normality with the Shapiro–Wilk test. Patient and tumor characteristics are reported descriptively and are shown as mean with standard deviation (SD) or median with quartiles as appropriate. Categorical parameters are shown as frequencies and proportions and were compared using contingency table analysis and the χ^2^ test. Comparison of continuous variables was performed with the student *t*-test for normally distributed variables or the Kruskal–Wallis test for not normally distributed variables.

OS was defined as the time interval from the date of diagnosis to the date of death or censoring date. Patients, who were lost to follow-up or without evidence of death, were censored with the date of the last follow-up. Median follow-up was calculated by means of the reversed Kaplan–Meier estimator and median OS was calculated by means of the Kaplan–Meier estimator and a confidence interval (CI) of 95%. Univariate potential prognostic predictors for survival were assessed using the log-rank test with a level of significance of 5% (chi-square *p* = 0.05), followed by multivariate Cox regression analysis to assess the independent prognostic value. A two-sided *p*-value < 0.05 was considered statistically significant. All methods and results are reported according to the STrengthening the Reporting of OBservational studies in Epidemiology (STROBE) guidelines [34].

## 3. Results

### 3.1. Patient Characteristics

**Figure 2:**
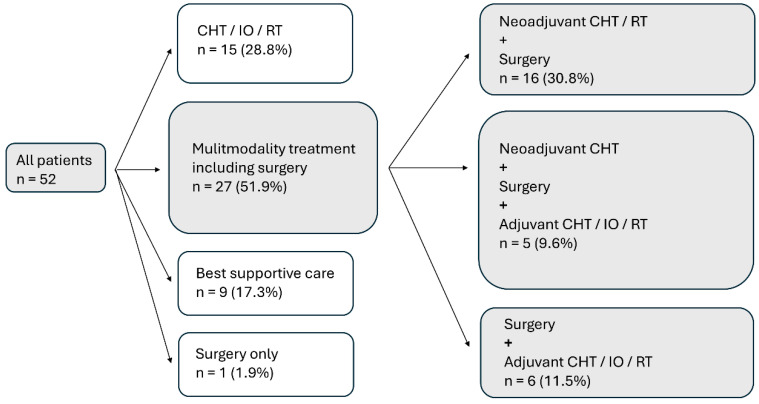
Treatment received by 52 patients with PM. PM, pleural mesothelioma; CHT, chemotherapy; IO, immunotherapy; RT, radiotherapy.

**Figure 3:**
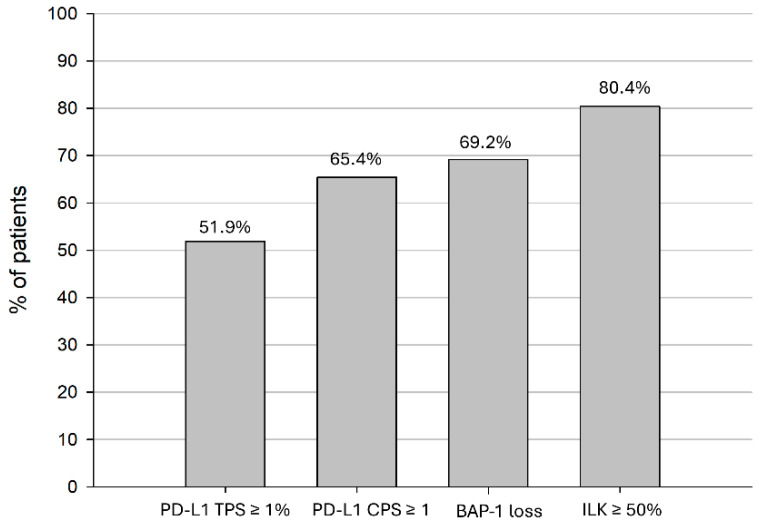
Expression of PD-L1 TPS and CPS, BAP-1 loss and ILK in patients with PM. CPS, combined positive score; PM, pleural mesothelioma; PD-L1, programmed cell death ligand 1; TPS, tumor proportion score.

In total, 52 patients diagnosed with PM between January 2010 and December 2021 were enrolled in the study. Patients were predominantly male (78.9%), and the median age at the time of diagnosis was 70 years. PM was more often found on the right side (67.3%). Most patients had an epithelioid histologic subtype (92.3%) and were diagnosed at stage III or IV (73.0%). Approximately half of the patients (51.9%) received MMT, 30.8% were treated with active oncologic treatment (CHT, IO, RT, RT-CHT or only surgery) and 17.3% received BSC. A total of 38 patients (73.1%) received CHT, 12 (23.1%) received IO and 8 (15.4%) received RT in the course of their treatment. Details of the various treatments can be found in Figure 2. Approximately half of patients (51.9%) had a PD-L1 TPS of ≥1%, and 65.4% had a PD-L1 CPS of ≥1. Seven patients (13.5%) who had a PD-L1 TPS < 1% showed a PD-L1 CPS ≥ 1. A loss of BAP1 was found in 69.2%, and 80.4% showed an ILK ≥ 50% (Figure 3). All patient characteristics can be seen in Table 1, and the expression of PD-L1, BAP-1 and ILK according to patient characteristics can be seen in Table 2.

### 3.2. Associations of Clinicopathological Parameters with the Expression of PD-L1, BAP-1 and ILK

Associations between clinicopathological parameters, including age, gender, laterality, histology, treatment modality, stage, and the expression of PD-L1, BAP-1 loss and ILK, were examined. Data were dichotomized into categories where necessary. For PD-L1 expression, cutoffs of ≥1% and ≥10% were used. For ILK expression, a cutoff of ≥50% as well as intensity (n = 50) dichotomized into weak (n = 27, 54%) versus moderate and strong (n = 23, 46%) was applied. A significant association was found between PD-L1 TPS ≥ 1% as well as PD-L1 TPS ≥ 10% and histologic subtype (epithelioid vs. non-epithelioid) (*p* = 0.045, *p* = 0.024), with PD-L1 TPS ≥ 1% and ≥ 10% being more frequent in non-epithelioid PMs. Moreover, a trend towards significance was observed between PD-L1 CPS ≥ 10 and histologic subtype (epithelioid vs. non-epithelioid) (*p* = 0.060), with more tumors of non-epithelioid subtype expressing PD-L1 CPS ≥ 10. Another significant association was seen between both PD-L1 TPS ≥ 1% and PD-L1 TPS ≥ 10% with stage (early vs. late) (*p* = 0.034, *p* = 0.036), as PD-L1 expression was more often expressed at a late PM stage. Furthermore, PD-L1 CPS ≥ 10 showed a significant association with stage (early vs. late) (*p* = 0.016), with more tumors expressing PD-L1 CPS ≥ 10 in the late stage. Additionally, ILK intensity and stage (early vs. late) were found to be associated (*p* = 0.032) with a weak ILK intensity being more often found in the late stage. 

### 3.3. Survival Analysis

Of 52 patients, 8 (15.4%) were alive at the end of the study. The median follow-up time was 55.6 (43.4; 109.7) months. The median OS of all patients was 16.9 (13.1; 25.2) months. The tumor stage significantly influenced OS (*p* = 0.002). Patients in the early stage showed a significantly longer median OS of 95.4 months compared to 15.3 months for patients in the late stage. Patients treated with MMT showed a longer OS (23.4 (15.3; 32.2)) than patients treated with active treatment (15.3 (9.2; 31.0)) or BSC (3.0 (0.6; 5.6)) (*p* = 0.001). An analysis of patients receiving CHT alone (n = 13) as an active treatment had a median OS of 21.3 (7.5; 31.0) months. Patients with a PD-L1 TPS ≥ 1% and CPS ≥ 1 showed numerically a lower OS of 15.3 (11.5; 24.4) vs. 20.0 (11.2; 34.9) and 15.3 (11.5; 25.4) vs. 20.0 (8.2; 32.2) months, respectively. The results of the univariate survival analysis for all patients can be seen in Table 3. In the multivariate Cox regression model, MMT and early stage were found to be independent prognostic factors for superior OS (MMT: HR 0.347, CI 0.13; 0.90, *p* = 0.029; stage: HR 4.989, CI 1.64; 15.13) *p* = 0.005) (Table 3).

Patients with a PD-L1 TPS ≥ 1% and CPS ≥ 1 showed numerically a lower OS of 15.3 (11.5; 24.4) vs. 20.0 (11.2; 34.9) and 15.3 (11.5; 25.4) vs. 20.0 (8.2; 32.2) months, respectively. Patients who received IO in the course of their treatment (n = 12) had a longer survival with 24.0 (14.6; NA) vs. 15.2 (11.2; 21.3) months.

Patients with BAP-1 loss presented with a longer survival of 20 (15.2; 28.1) months than BAP-1 positive patients with 11.3 (5.6; 31.0) months (*p* = 0.153). An analysis of all patients receiving CHT in the course of their disease (n = 38) showed a shorter survival in BAP-1 positive patients with 15.8 (6; 35.6) vs. 23.4 (15.3; 28.3) months. The combination of CPS ≥ 1% or TPS ≥ 1% and BAP-1 positivity was associated with worse survival (*p* = 0.045, *p* = 0.059, respectively).

**Figure 4:**
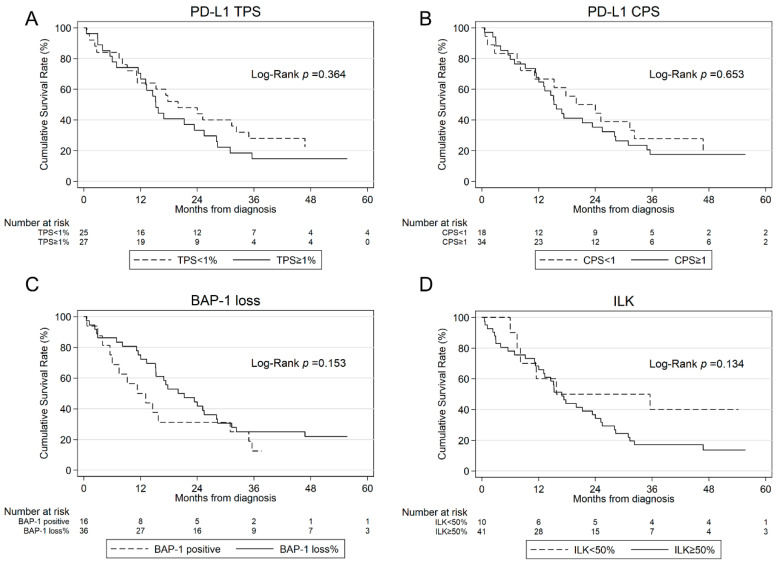
Kaplan–Meier estimates for OS according to PD-L1 TPS (**A**), PD-L1 CPS (**B**), BAP-1 loss (**C**) and ILK (**D**) in all patients. OS, overall survival; PD-L1, programmed cell death ligand 1; TPS, tumor proportion score; CPS, combined positive score; ILK, integrin-linked kinase.

Patients with ILK expression showed no difference in survival time with 16.9 (12.1; 24.0) vs. 15.8 (6.0; NA) months. The combination of ILK expression ≥50% and PD-L1 CPS ≥ 1% or TPS ≥ 1% was associated with worse survival (*p* = 0.019, *p* = 0.023, respectively). Kaplan–Meier curves for PD-L1 TPS and CPS, BAP-1, and ILK are illustrated in Figure 4. 

### 3.4. Patients with Multimodality Treatment Including Surgery

**Figure 5:**
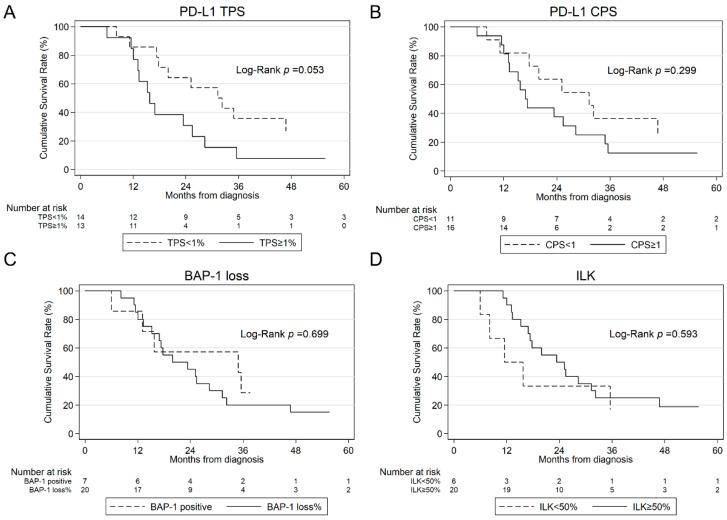
Kaplan–Meier estimates for OS according to PD-L1 TPS (**A**), PD-L1 CPS (**B**), BAP-1 loss (**C**) and ILK (**D**) in 27 patients with PM undergoing MMT. OS, overall survival; PD-L1, programmed cell death ligand 1; TPS, tumor proportion score; CPS, combined positive score; ILK, integrin-linked kinase.

The median OS of the subgroup of 27 patients with PM treated with MMT was 23.4 (15.3; 32.2) months. In patients treated with MMT, similarly to the whole cohort, patients in the early stage had a significantly longer survival with 95.4 (13.3; NA) months than patients at a late stage with 17.4 (12.1; 25.4) months (*p* = 0.014). Furthermore, patients undergoing MMT with PD-L1 TPS ≥ 1% had a reduced OS of 15.8 (12.1; 25.4) vs. 31.3 (17.4; 95.4) months in PD-L1 negative patients (*p* = 0.053). Those who received IO in the course of their treatment (n = 5) had a longer survival with 46.8 (15.3; NA) vs. 17.8 (13.1; 31.3) months. Interestingly, a lower survival in patients with MMT with BAP-1 loss with 34.9 (6.0; NA) vs. 20.0 (13.3; 31.3) months was observed. However, the number of BAP-1-positive patients was low, and the confidence interval was large. MMT patients with ILK expression showed a numerically longer OS with 23.4 (15.3; 32.2) vs. 11.5 (6.0; NA) months, however, without reaching significance (*p* = 0.593). All results of the univariate analysis of 27 patients with PM undergoing MMT can be found in Table 4 and Kaplan–Meier curves for PD-L1 TPS and CPS, BAP-1 and ILK are shown in Figure 5. A multivariate Cox regression analysis of potential prognostic factors (*p* < 0.1) showed no significant differences (Table 4).

## 4. Discussion

PM is a rare disease; however, its dismal survival prognosis and limited treatment options demonstrate an unmet medical need for prognostic and predictive markers. This descriptive analysis of clinical characteristics and biomarkers in a European PM cohort provides comprehensive data on expression patterns and prognostic roles of BAP-1, ILK and PD-L1.

So far, MMT consisting of a combination of surgical intervention and neoadjuvant or adjuvant CHT with or without RT has been reported to be a viable treatment option for patients in the setting of resectable disease [33,35]. However, recently, the MARS 2 study comparing surgery followed by CHT with CHT alone revealed a longer median survival for patients receiving CHT alone with 24.8 vs. 19.3 months with more serious adverse events for patients in the surgery cohort [36]. Our results show an independent significant survival benefit (HR 0.347) with a median survival of 23.4 months in the MMT-treated cohort in our study, and we report a median survival times of 21.3 months in patients receiving CHT alone. Another important study is CheckMate 743, assessing first-line IO with nivolumab plus ipilimumab in unresectable PM. Combination of nivolumab, a PD-1 antibody, with ipilimumab, a CTLA-4 antibody, has shown improvements of 18.1 months vs. 14.1 months with platinum plus pemetrexed chemotherapy [21,22]. These conflicting data furthermore highlight the unmet need for strong predictive biomarkers in order to tailor individualized and potentially radical treatment to selected patients with PM.

The potential of PD-L1 expression of a tumor as a predictive biomarker for clinical response to IO has been shown in several tumors including non-small cell lung cancer (NSCLC) [37]. However, in PM studies comparing prognostic factors without the treatment option of IO, PD-L1 has been reported to be associated with rather worse survival [16,20]. In our study, we observed rather high rates of PD-L1 positive (51.9%) tumor and high proportion of patients with PD-L1 expressions of 10% or higher (26.9%). These findings are higher than in other European cohorts, but rates of 42.4% have been reported in a comparable study in South America [16,19,20]. A comparison of PD-L1 expression patterns with clinicopathological characteristics showed that PD-L1 expression was associated with non-epithelioid histologic subtypes. This is in accordance with previous studies reporting more often sarcomatoid or biphasic subtypes to be PD-L1 positive [19,26,38]. Additionally, we found a correlation between advanced tumor stage and PD-L1 expression. This association was not found in previous studies; however, this may be due to the fact that studies assessed PD-L1 expression association mainly for patients in advanced stage [19,38]. Nevertheless, it has been shown that the PD-L1 expression of tumor-associated macrophages increases with disease stage in primary human cancers and over time in mouse models [39]. While no threshold PD-L1 expression has been defined for the treatment response prediction or survival probability in PM, many previous studies have applied a cut-off level of 1% [15,16,20]. Similarly, in this study, we correlated survival with PD-L1 using a cut-off level of 1% and found in accordance with previous studies a worse survival in patients with PD-L1 expression of 15.3 vs. 20.0 months irrespective of treatment [16,20]. Moreover, this survival disadvantage was significant, with 15.8 vs. 31.3 months in the cohort of patients treated with MMT. Nevertheless, in multivariate analysis, it did not prove to be an independent prognostic factor. Many previous studies reporting PD-L1 expression to be correlated with a survival disadvantage did not include or actively exclude patients receiving IO [15,16,19,20]. In our study, 12 patients (23.1%) received IO in the course of their treatment, of which 5 patients (41.7%) received it as part of MMT. All patients receiving IO showed a longer survival with 24.0 vs. 15.2 months. This survival benefit was even more distinctive with 46.8 vs. 17.8 months for patients who received IO as part of MMT, suggesting the benefits of IO as a novel treatment option.

Another marker that has been reported to help predict prognosis and to individualize treatment is BAP-1, a tumor suppressor involved in homologous recombination-mediated DNA repair and cell death [40]. Former studies have reported that BAP-1 loss leads to an increase in OS in patients with PM and in sensitivity to DNA-damaging agents such as platinum CHT, however, this matter is controversial [40,41]. Approximately 7.7% of patients with PM with familial PM carrying germline BAP-1 mutations and in sporadic PM nuclear BAP-1 expression was shown to be lost in 60.3 to 67.1% [10,42,43]. Similarly, we report loss of BAP-1 expression in 69.2% of patients. In the overall study cohort, patients with BAP-1 loss showed numerically a longer survival with 20.0 vs. 11.3 months in BAP-1 positive patients. Most studies assessing the prognostic role of BAP-1 expression evaluate the prognostic value in patients treated with platinum-based CHT only [41,43,44]. Louw et al. analyzed two PM cohorts with a total of 348 patients treated with a combination of platinum and pemetrexed therapy and found significant survival benefits for patients with BAP-1 loss in both cohorts with 20.1 and 19.6 vs. 7.3 and 11.1 months [43]. In accordance, we report longer survival times in 38 patients with BAP-1 loss who received CHT in the course of their treatment. In contrast to Louw et al., we report an longer survival time of BAP-1 positive patients receiving treatment vs. best supportive care only (median OS of 3.0 vs. 15.8 months) [43].

Another potential biomarker that was assessed for its prognostic value in this study is ILK. As an important link to chronic inflammation, ILK has been correlated with tumor progression patterns and worse survival through its interaction with the extracellular matrix in other tumor types including NSCLC [45]. While ILK is not expressed in normal mesothelial cells and lung parenchyma, it was found to be increased in cells of patients with PM [8]. Furthermore, serum analyses showed an increase in ILK expression between patients with asbestos exposure and patients with PM [46]. In our study cohort, ILK was highly expressed in 80.4%. However, ILK expression did not influence patients’ survival. In MMT treated patients ILK expression of 50% or higher seemed to be potentially protective with a median survival of 23.4 vs. 11.5 months. Interestingly, similar results were found in a study by Schramm et al. assessing ILK in survival data of 128 patients with PM. In their study, high ILK was associated with a significantly longer survival (13.2 vs. 10.7 months) [24]. In our cohort, a weak intensity of ILK was significantly more often found in a late tumor stage, whereas moderate or strong intensity was balanced between an early and late stage. This is especially noteworthy, as the tumor stage was the only independently significant factor in our analyses. However, to determine whether a strong ILK intensity or a high ILK expression is favorable for prognosis further research is needed.

The combination of CPS ≥ 1% and BAP-1 positivity was associated with worse survival (*p* = 0.045) in our patients. Furthermore, the combination of CPS ≥ 1% or TPS ≥ 1% and ILK expression ≥50% resulted in significant poorer OS (*p* = 0.019, *p* = 0.023, respectively). Given the limited number of cases, these results should be interpreted with caution, as the small sample size may limit the generalizability of the findings.

Although multiple potential prognostic markers have been assessed in this study, the only independent predictor for OS besides MMT was tumor stage. This finding is, of course, consistent with most other studies [9,16].

This study is limited by its retrospective nature and the limited number of patients included due to the rarity of the disease. The long retrospective character led to many different therapeutic approaches and treatment regimens. Nevertheless, the inclusion of various treatments including IO enabled us to assess prognostic values and survival patients more thoroughly.

In conclusion, our exploratory analysis demonstrates high rates of PD-L1, BAP-1, and ILK expression in PM patients. It also shows that patients with PM with PD-L1 expression had survival disadvantages. Additionally, we report significant survival advantages for patients receiving MMT and found numerically longer survival, especially in patients who received IO as part of their MMT treatment. Patients with BAP-1 loss showed numerically a survival advantage. Our results indicate that treatment decisions in PM might be individualized based on biomarker expression patterns.

## Figures and Tables

**Table 1:** Patient characteristics.

Characteristics	n = 52
Age, years	
Median (range)	70 (41–85)
Age groups, n (%)	
<65	13 (25.0)
≥65	39 (75.0)
Sex, n (%)	
Male	41 (78.9)
Female	11 (21.2)
Laterality, n (%)	
Right	35 (67.3)
Left	17 (32.7)
Histology, n (%)	
Epithelioid	48 (92.3)
Biphasic	3 (5.8)
Sarcomatoid	1 (1.9)
Treatment, n (%)	
Multimodality treatment	27 (51.9)
Active therapy	16 (30.8)
Best supportive care	9 (17.3)
Stage (n = 37), n (%)	
Early (I/II)	10 (27.0)
Late (III/IV)	27 (73.0)
PD-L1, n (%)	
TPS < 1%	25 (48.1)
TPS ≥ 1%	27 (51.9)
PD-L1, n (%)	
TPS < 10%	38 (73.1)
TPS ≥ 10%	14 (26.9)
PD-L1, n (%)	
CPS < 1	18 (34.6)
CPS ≥ 1	34 (65.4)
PD-L1, n (%)	
CPS < 10	35 (67.3)
CPS ≥ 10	17 (32.7)
BAP1, n (%)	
Loss	36 (69.2)
Positive	16 (30.8)
ILK (n = 51), n (%)	
ILK < 50%	10 (19.6)
ILK ≥ 50%	41 (80.4)

The percentage may not equal to 100 due to rounding. PD-L1, programmed cell death ligand 1; TPS, tumor proportion score; CPS, combined positive score; ILK, integrin-linked kinase.

**Table 2:** Expression of PD-L1, BAP-1 and ILK according to patient characteristics.

	PD-L1 TPS ≥ 1% (n = 52)	PD-L1 CPS ≥ 1 (n = 52)	BAP1 Loss (n = 52)	ILK ≥50% (n = 51)
Age, years, n (%)				
<65	6 (46.2)	9 (69.2)	11 (84.6)	10 (76.9)
≥65	21 (53.9)	25 (64.1)	25 (64.1)	31 (81.6)
Gender, n (%)				
Male	21 (51.2)	26 (63.4)	29 (70.7)	32 (80.0)
Female	6 (54.6)	8 (72.7)	7 (63.6)	9 (81.8)
Laterality, n (%)				
Right	16 (45.7)	23 (65.7)	24 (68.6)	27 (79.4)
Left	11 (64.7)	11 (64.7)	12 (70.6)	14 (82.4)
Histology, n (%)				
Epithelioid	23 (47.9)	30 (62.5)	33 (68.8)	39 (83.0)
Biphasic	3 (100)	3 (100)	3 (100)	2 (66.7)
Sarcomatoid	1 (100)	1 (100)	0 (0)	0 (0)
Treatment, n (%)				
Multimodality	13 (48.2)	16 (59.3)	20 (74.1)	20 (76.9)
Active treatment	9 (56.3)	13 (81.3)	11 (68.8)	12 (75.0)
Best supportive car	5 (55.6)	5 (55.6)	5 (55.6)	9 (100)
Stage, n (%)				
Early (I/II)	2 (20.0)	5 (50.0)	8 (80.0)	9 (90.0)
Late (III/IV)	16 (59.3)	19 (70.4)	19 (70.4)	20 (76.9)
No data	9 (64.3)	10 (71.4)	8 (57.1)	11 (78.6)

The percentage may not equal to 100 due to rounding. PD-L1, programmed cell death ligand 1; TPS, tumor proportion score; CPS, combined positive score; ILK, integrin-linked kinase.

**Table 3:** Univariate survival analysis and multivariate Cox regression model for OS adjusted for clinicopathological variables for 52 PM patients.

N = 52	No. of Patients	Median OS (in Months) (95% CI)	*p*-Value	Multivariate Cox Regression	
				HR (95% CI)	*p*-Value
Age, years					
Age groups					
<65	13	15.8 (11.2; 25.2)	0.674	0.965 (0.92; 1.01)	0.136
≥65	39	20.0 (12.1; 28.3)			
Gender					
Male	41	16.9 (11.5; 24.0)	0.723	0.536 (0.18; 1.57)	0.254
Female	11	25.2 (3.0; 35.6)			
Laterality					
Right	35	17.8 (11.5; 31.0)	0.352	0.975 (0.40; 2.38)	0.956
Left	17	15.3 (7.5; 28.1)			
Histology					
Epithelioid	48	16.9 (12.1; 25.2)	0.684	1.997 (0.32; 12.40)	0.458
Non-epithelioid	4	13.3 (3.0; NA)			
Treatment					
Multimodality treatment	27	23.4 (15.3; 32.2)	**0.001**	0.347 (0.13; 0.90)	**0.029**
Active treatment	16	15.3 (9.2; 31.0)			
Best supportive care	9	3.0 (0.6; 5.6)			
Stage					
Early (I/II)	10	95.4 (2.4; NA)	**0.002**	4.989 (1.64; 15.13)	**0.005**
Late (III/IV)	27	15.3 (11.3; 21.3)			
No data *					
PD-L1					
TPS < 1%	25	20.0 (11.2; 34.9)	0.364	1.366 (0.54; 3.46)	0.511
TPS ≥ 1%	27	15.3 (11.5; 24.4)			
PD-L1					
TPS < 10%	38	15.8 (11.5; 25.4)	0.861		
TPS ≥ 10%	14	16.9 (6.0; 35.6)			
PD-L1					
CPS < 1	18	20.0 (8.2; 32.2)	0.653		
CPS ≥ 1	34	15.3 (11.5; 25.4)			
PD-L1					
CPS < 10	35	15.3 (11.3; 25.2)	0.775		
CPS ≥ 10	17	21.3 (6.9; 34.9)			
BAP-1					
Loss	36	20.0 (15.2; 28.1)	0.153	0.804 (0.28; 2.28)	0.683
Positive	16	11.3 (5.6; 31.0)			
ILK					
ILK < 50%	10	15.8 (6.0; NA)	0.134	0.874 (0.24; 3.14)	0.837
ILK ≥ 50%	41	16.9 (12.1; 24.0)			
No data *					

OS, overall survival; PD-L1, programmed cell death ligand 1; TPS, tumor proportion score; CPS, combined positive score; ILK, integrin-linked kinase; HR, hazard ratio; * excluded from analysis. Significant *p* values are highlighted bold.

**Table 4:** Survival analysis and multivariate Cox regression model for OS adjusted for clinicopathological variables for 27 PM patients treated with MMT.

n = 27	No. of Patients	Median OS (in Months)	*p*-Value	Multivariate Cox Regression	
				HR (95% CI)	*p*-Value
Age, years					
Age groups					
<65	10	16.9 (11.2; 31.3)	0.807	0.988 (0.93; 1.05)	0.674
≥65	17	25.4 (13.1; 35.6)			
Gender					
Male	19	17.8 (13.3; 32.2)	0.317	0.358 (0.10; 1.32)	0.123
Female	8	28.3 (12.1; NA)			
Laterality					
Right	20	23.4 (15.3; 46.8)	0.097	1.121 (0.39; 3.22)	0.832
Left	7	15.8 (11.2; 28.3)			
Histology					
Epithelioid	25	23.4 (15.8; 32.2)	0.833	0.953 (0.09; 10.11)	0.968
Non-epithelioid	2	13.3 (13.3; NA)			
Stage					
Early (I/II)	7	95.4 (13.3; NA)	**0.014**	2.845 (0.76; 10.62)	0.120
Late (III/IV)	19	17.4 (12.1; 25.4)			
No data *	1				
PD-L1, n (%)					
TPS < 1%	14	31.3 (17.4; 95.4)	**0.053**	2.274 (0.75; 6.94)	0.149
TPS ≥ 1%	13	15.8 (12.1; 25.4)			
PD-L1, n (%)					
TPS < 10%	22	25.2 (15.3; 34.9)	0.179		
TPS ≥ 10%	5	16.9 (6.0; NA)			
PD-L1, n (%)					
CPS < 1	11	31.3 (11.2; 95.4)	0.299		
CPS ≥ 1	16	16.9 (13.1; 28.3)			
PD-L1, n (%)					
CPS < 10	20	25.2 (13.3; 46.8)	0.226		
CPS ≥ 10	7	17.4 (6.0; 34.9)			
BAP-1, n (%)					
Loss	20	20.0 (13.3; 31.3)	0.699	1.118 (0.27; 4.67)	0.879
Positive	7	34.9 (6.0; NA)			
ILK, n (%)					
ILK < 50%	6	11.5 (6.0; NA)	0.593	0.553 (0.11; 2.74)	0.467
ILK ≥ 50%	20	23.4 (15.3; 32.2)			
No data *	1				

MMT, multimodality treatment; OS, overall survival; PD-L1, programmed cell death ligand 1; TPS, tumor proportion score; CPS, combined positive score; ILK, integrin-linked kinase; HR, hazard ratio; * excluded from analysis. Significant *p* values are highlighted bold.

## Data Availability

The data presented in this study are available on reasonable request from the corresponding author. The data are not publicly available due to the valid European General Data Protection Regulations.

## Abbreviations

BAP-1	Breast cancer gene 1-associated protein
CTLA-4	Cytotoxic T-lymphocyte-associated protein 4
EORTC	European organization for research and treatment of cancer
EPD	Extended pleurectomy/decortication
EPP	Extrapleural pneumonectomy
FFPE	Formalin-fixed paraffin-embedded
HR	Hazard Ratio
ILK	Integrin-linked kinase
IO	Immunotherapy
MMT	Multimodality treatment including surgery
NSCLC	Non-small cell lung cancer
OS	Overall survival
P/D	Pleurectomy/decortication
PD-L1	Programmed death ligand 1
RT	Radiotherapy
RT-CHT	Combined chemoradiotherapy
TPS	Tumor proportion score
